# Book Reviews

**Published:** 1916-12

**Authors:** 


### BOOK REVIEWS

Books mentioned may be inspected at and ordered through this office. So far as possible, books received in any month will be reviewed in the issue of the second month following.

**Mortality Statistics,** 1914, issued by the U. S. Bureau of the Census, is acknowledged with thanks.

In contrast with the recent epidemic, it is interesting to note that acute anterior poliomyelitis caused only 620 deaths in the entire registration area whose population is about 6½ times that of N. Y. State. 397 were in males, 323 in females; 220 and 196, respectively, or a total of 416 (almost exactly 2/3) were in children under 5. It is significant, however, that from this point on, the decline was quite gradual, 4 deaths being listed for the quinquennium 75-79 years. Measles causes about three quarters of its fatalities under 5, and scarlet fever a little less than half while both show the same gradual decline with increasing age and persistence of fatalities due to advanced ages. Whooping cough causes 97.3% of its fatalities under 5, shows, therefore, a more marked decline after this age but also persists to advanced ages. These are, we believe, the only infections that can be considered characteristically children's diseases. When we analyse the meaning of this expression, it implies semelincidence, i.e. practically life-long immunity after an attack, a failure of hereditary immunity unless very recently after a maternal attack, so that "children's diseases" are those to which the whole human race is more or less susceptible and thus, they occur in children merely in accordance with the law of chance. Small pox, before the use of vaccination, was equally a children's disease and the explanation of the change in this respect is too obvious to require discussion. There was no large epidemic of poliomyelitis in 1914 and the statistics are all the more enlightening for that reason. They show that this disease, to which the term infantile has been applied, occurs throughout life, like other general infections; that it is not significantly more infantile than scarlet fever, somewhat less so than measles, and decidedly less so than whooping cough. It is a proper logical principle, to suspect the reality, as an independent and essential condition, of anything that is exceedingly rare and this principle has been applied by many to poliomyelitis. But we note that, in an off-year, this disease shows a mortality of 1/7-1/10 of the disease generally accepted as specific infections to which the human race is markedly susceptible. Owing to the differences in mortality, the ratio of incidence is, of course, much smaller but this fact operates in the opposite direction also, by removing the constant reminder of the existence of a disease, by case histories. Thus, the mere study of statistics, shows that poliomyelitis is by no means out of analogy with the other so-called "children's diseases."

---

**Physiologic Chemistry.** Albert P. Mathews, Ph. D., Chicago. Wm. Wood & Co. 1040 pages, illustrated, $4.25, 2d edition.

We have previously reviewed favorably the first edition. We want to commend this book especially to the general practitioner, as throwing light on many phases of bodily function. While strictly a scientific text book, the author describes many clinical methods of analysis which can be carried out in a simple laboratory, and uses an almost popular style in describing the results of metabolism experiments, ferment action, etc., so that the deductions from highly technical chemic research are available to all. The thoroughness of the table of contents and the index, and the copious bibliographies also add to the value of the work for reference. As a laboratory guide, the book needs no introduction, but the broad conception of vital processes and the elucidation of many vaguely understood matters of diagnosis and therapeutics dependent upon such a conception are points which the physician might not realize.

---

**Diseases of Infants and Children.** Henry Dwight Chapin, A. M., M. D., and Godfrey Pisek, M. D., Sc. D., N. Y. Wm.

Wood & Co., N. Y. 578 pages, 179 cuts, 12 colored plates. $3.25, 3d edition.

This work contains numerous tables and diagrams, of which latter the ones representing graphically the weights and measurements of infants are especially novel. While hygiene, feeding and general methods of examination, diagnosis and management are well treated, the work is mainly what it purports to be and what some text books with similar titles fail to be through undue attention to generalities. That is, it really discusses in detail, the diseases of infants and children.

---

**Vaccine Therapy in General Practice.** G. H. Sherman, M. D., Detroit, author and publisher. 3d edition, 523 pages, $2.50.

This is an exhaustive, practical treatise, covering not only diseases commonly recognized as infectious, but those ultimately due to infection, as diabetes (inflammatory reaction in the insulae of the pancreas), goitre, etc., as well as various inflammatory conditions, as of the respiratory passages, commonly recognized as infectious but not in the ordinary sense specific. From experiments by Vaughan and others, it is concluded that toxic phenomena of fatal degree would require about 450 m. g. of bacterial proteins, corresponding to about 2 quarts of average bacterial vaccine. The author has never serious anaphylactic phenomena. The negative phase is important only in tuberculosis. Hence, generally speaking, vaccines are harmless, even if not successful. Autogenic vaccines are considered as theoretically rather than practically superior to stock vaccines, and there are flaws even in the theoretic advantages of the former. At the same time, the author is not a biased enthusiast, but speaks conservatively of the general method, and candidly reports failures and discusses the probabilities of success and failure in different types of cases.

---

**Ethnology of the Tewa Indians.** Wm. M. Robbins, John Peabody Harrington and Barbara Freire Marreco, Bureau of American Ethnology, Bulletin 55.

This is an extremely interesting compend, including many references to language, customs and rites.

---

**Institution Quarterly.** Vol. 7 No. 3, Sept. 30, 1916. Issued jointly by the State Board of Administration, State Charities Commission and State Psychopathic Institute of Illinois. Editor-in-chief, A. L. Bowen, Springfield.

The present issue, aside from administrative and economic details, deals mainly with sociologic problems.

---

**Practical Bacteriology, Blood Work and Animal Parasitology.** E. R. Stitt, A. B., Ph. G., M. D., Medical Director U. S. Navy. P. Blakiston's Son & Co., Philadelphia. 497 pages, 4 plates, 115 other illustrations, $2.00 (4th edition).

This work, already favorably known through earlier editions, is condensed in style and arrangement, but as the word condensed as applied to books, is often used to imply abbreviation and omission, it might be better to say that the contents of this book are compressed. The pictures are small and not very good yet, somehow, they illustrate better than more imposing photographic reproductions, and without being semi-diagrammatic either. There is an originality of treatment of the whole subject which makes a very favorable impression and which is difficult of analysis. Perhaps this is because the author knows just how far condensation may safely be carried without omission of important details and without rendering a description inadequate; when to illustrate and when to describe in words; when to use a tabular classification and when this method stretches facts; when a cross reference or allusion to general principles suffices and when a particular description is necessary.

**The Genus Hippochaete in North America, North of Mexico.** Oliver Atkins Farwell, Detroit. Reprint from Memoirs of N. Y. Botanical Garden.

These plants are named from their resemblance to a horse's mane, and are related to the equisetum or horse tail rush, used medicinally.

---

**Muscle Training in the Treatment of Infantile Paralysis.** Wilhelmine G. Wright, Boston. Published by Ernest Gregory, Boston. 2d edition, 30 pages, paper cover, 25 cents.

---

**How to Live.** Irving Fisher of Yale University and Eugene Lyman Fisk, N. Y., Director of Hygiene of the Life Extension Institute. Funk & Wagnalls Co., N. Y. 8th edition, 345 pages, $1.00.

This is a popular book on personal hygiene authorized by the Institute. It differs from the ordinary type of hygienic text book in presenting statistics gathered from various sources, and in considering the individual as a member of society rather than a unit. Thus the trend of the work is largely sociologic. Considerable attention, therefore, is logically devoted to eugenics and the work, though popular, is scientific.

---

**Human Physiology.** Albert P. Brubaker, A. M., M. D., Philadelphia, P. Blakiston's Son & Co. 5th edition, 776 pages, 359 illustrations and 1 plate in color, $3.00.

It may not be out of place to call attention to the surprising cheapness of this work, merely on a basis of bulk. Moreover, while not unduly crowded, nor in fine print, the arrangement of the work gives the maximum of reading per page. Thus, in extent, the work may fairly rank with the major treatises in this or any other branch of medicine. It is in no sense a brief or condensed text book. The reputation of the author and of his book—remembering that these are by no means necessarily cognate—are already of the highest rank. Metabolism experiments, including those in calorimetry, the internal secretions and the correlation of clinical chemistry and microscopy with scientific study of such divisions of the work as digestion, circulation and the blood, urinary organs, etc., show a considerable advance since the fourth edition. An appendix dealing with physiologic apparatus, which will be somewhat disappointing to anti-vivisectionists, is added to the work.

---

**Pharmacology and Therapeutics.** H. C. Wood, Jr., M. D., Philadelphia. J. B. Lippincott Co., Philadelphia. 2d edition, 455 pages, $4.00.

Like his illustrious father, whom the reviewer was so fortunate as to have among his teachers, Dr. Wood follows the method of classifying drugs as to physiologic action but, comparing the present classification with that in vogue 30 years ago, one notes a considerable advance in scientific pharmacology, especially noteworthy in the more general grouping of classes of drugs. The preliminary discussion of pharmacology is similarly, familiar and, at the same time, shows progress. It is perhaps a personal bias that leads to the question as to whether the metric system is "rarely employed" in this country generally although it is, unfortunately, used in the minority of cases, even after 50 years of legal establishment. Ionization is well discussed, with one regard to its present status in practical pharmacology. It occurs to us that the time is ripe for a general and subclassified discussion of the effect of drugs on the reaction of fluids, both from the theoretic and the practical standpoint, as well as of adsorbents, as a general class. Also the solvent effects of drugs upon calculi should be generally discussed, even from the negative, sceptic standpoint. This is, at least, a live issue with the profession as a whole, and many who do not believe that there exist solvent drugs, which adds greatly to the comprehension of the subject. So, too, his personal studies in scientific pharmacology and the correlation of experiment and experience, enhance the value of the book. Such a work as this is, unconsciously perhaps, a protest against the recent elimination of therapeutics from the strictly medical curriculum, by at least one prominent institution.

---

**Patience Worth, a Psychic Mystery.** Casper S. Yost, published by Henry Holt & Co., N. Y.

This book has already attracted much attention from medical journals. It purports to be the communications, by ouija board, of a lady who is rather vague as to the period of her sojourn on earth but who was pinned down to the date 1649, on one occasion. The communications consist of epigrammatic, tart and interesting conversations, stories of mediaeval life and some poetry. As is characteristic of language in general, the last does not show much difference in idiom to modern compositions, the stories are decidedly archaic and—as is also to be expected in literature—mainly set by implication at a considerably earlier period than the seventeenth century, while the conversational idioms and forms impress us as being a good deal more different to modern English than would be expected. The claim that Patience Worth, whoever or whatever she may be, steers clear of anachronisms of allusion to modern environment, seems justified and the differences in style with reference to antiquity of language, among the conversations, poems and prose stories, is not necessarily an anachronism. Without claiming to be a critical language scholar, we are rather dubious as to whether English people of the middle of the seventeenth century used the peculiar, at times almost unintelligible idioms, confused the second person pronouns as to number and case and, in general, spoke so differently from present standards. While granting the interest of the book, as a literary production, we cannot, even under the guidance of the editor, enthuse to the degree constantly demanded, as to the excellence of the stories and poems. Without in any way disputing the sincerity of the transcriber from the ouija board, and the participants in the reception of the messages in St. Louis, we beg to retain more of the Missourian spirit than they themselves.

---

**Secrets of the German War Office.** Karl Graves.

Some months ago, we reviewed this book, very sceptically.

Recent events, fully presented in the newspapers, confirm our impression that the book was to be read simply for its literary interest.

---

**Medical Record Visiting List 1917.** Wm. Wood & C., N. Y.

This is issued in 3 regular sizes, for 30 patients a week, dated or undated, $1.25; for 60, dated or undated, $1.50; for 90, dated only, $2.00. Extra quality lists are also available at prices ranging from $2.50 to $4.00 and fillers for the leather wallets can also be purchased at 30 to 50 cents for 6 months. This Visiting list is standard through many years' use, contains a vast amount of valuable information for emergent reference and is almost perfectly adapted to the needs of any practitioner.

---

**Poverty and Health,** 39th Annual Report of the Charity Organization Society of Buffalo inclosing as a separate pamphlet, TEN TALES (illustrated) by Frederick Almy, Secretary, which present examples of the work of the Society in clinical form. We bespeak the careful reading of these pamphlets by the medical profession.

